# MicroRNA regulation of the MRN complex impacts DNA damage, cellular senescence, and angiogenic signaling

**DOI:** 10.1038/s41419-018-0690-y

**Published:** 2018-05-24

**Authors:** Cristina Espinosa-Diez, RaeAnna Wilson, Namita Chatterjee, Clayton Hudson, Rebecca Ruhl, Christina Hipfinger, Erin Helms, Omar F. Khan, Daniel G. Anderson, Sudarshan Anand

**Affiliations:** 1 Department of Cell, Developmental and Cancer Biology, Department of Radiation Medicine, Oregon Health and Sciences University (OHSU), 3181 SW Sam Jackson Park Road, Portland, OR 97239 USA; 2 Department of Chemical Engineering, Institute for Medical Engineering and Science, David H Koch Institute for Integrative Cancer Research, Massachusetts Institute of Technology, Cambridge, MA 02139 USA

## Abstract

MicroRNAs (miRs) contribute to biological robustness by buffering cellular processes from external perturbations. Here we report an unexpected link between DNA damage response and angiogenic signaling that is buffered by a miR. We demonstrate that genotoxic stress-induced miR-494 inhibits the DNA repair machinery by targeting the *MRE11a*-*RAD50*-*NBN* (MRN) complex. Gain- and loss-of-function experiments show that miR-494 exacerbates DNA damage and drives endothelial senescence. Increase of miR-494 affects telomerase activity, activates p21, decreases pRb pathways, and diminishes angiogenic sprouting. Genetic and pharmacological disruption of the MRN pathway decreases VEGF signaling, phenocopies miR-494-induced senescence, and disrupts angiogenic sprouting. Vascular-targeted delivery of miR-494 decreases both growth factor-induced and tumor angiogenesis in mouse models. Our work identifies a putative miR-facilitated mechanism by which endothelial cells can be insulated against VEGF signaling to facilitate the onset of senescence and highlight the potential of targeting DNA repair to disrupt pathological angiogenesis.

## Introduction

Accumulation of DNA damage can overwhelm the repair machinery and lead to senescence^1^. Endothelial senescence leads to progressive damage and deterioration of cellular structure and function over time^2^. Two major pathways of senescence in endothelial cells (ECs) are replicative senescence and stress-induced premature senescence (SIPS). Replicative senescence is one of the hallmarks of aging and is associated to telomere shortening. SIPS is triggered by external stimuli, including oxidizing agents and radiation, both of which can induce DNA damage and cell cycle arrest.

Recent studies indicate that DNA repair proteins in ECs have an inherently pro-angiogenic role^3,4^. For example, ATM deficiency decreases tumor angiogenesis while enhancing the anti-angiogenic action of vascular endothelial growth factor (VEGF) blockade. ATM can also function as a redox sensor independent of its DNA damage repair (DDR) function and regulate oxidative stress responses^5^ and was recently implicated as a driver of cellular senescence^6^. Similarly, global or EC-specific deletion of the histone H2AX in mice results in a substantial decrease of pathological angiogenesis in proliferative retinopathy, hindlimb ischemia, and tumor angiogenesis. These findings suggest that key regulators of DDR modulate pathological angiogenesis.

We screened for miR regulators of DDR in ECs and identified a group of seven miRs that were induced in common across different modes of DNA damage and oxidative stress^7^. We showed that the most robustly induced miR, miR-103, induced EC death by targeting the three prime exonuclease (TREX) pathway. In this study, we elucidate how two other miRs in our signature miR-494, and to a lesser extent, miR-99b, decrease DDR in ECs. Complementary to the miR-103-induced cell death, miR-494 enhances DNA damage leading to cellular senescence and decreased angiogenesis. We also demonstrate that miR-494-mediated EC DNA damage and its downstream effects are largely due to its downregulation of the Mre11a-*RAD50*-*NBN* (MRN) complex.

MRN complex acts as a sensor of DNA double-strand breaks (DSBs), initiating homologous recombination or non-homologous-end-joining pathway. Once MRN detects DSBs, it activates and recruits DNA damage response proteins such as ATM^8,9^. Interestingly, MRN is also associated with telomere maintenance, playing a role in the formation and disassociation of the t-loops^10,11^. The MRN complex relationship with aging and cell senescence has been described^12,13^, however its role in ECs and pathological neovascularization is unclear. Similarly, while several miRs have been shown to be involved in EC senescence^2,14,15^, proliferation, viability, and migration^16–18^, our work identifies a novel link between DDR-driven induction of miR-494, miR-99b, the MRN complex, EC senescence, and angiogenesis. Our results indicate that altering the DDR in ECs has a functional effect on angiogenesis possibly by activating a cellular senescence program.

## Results

### DNA damage induces miR-494 and miR-99b in vitro and in vivo

**Fig. 1:**
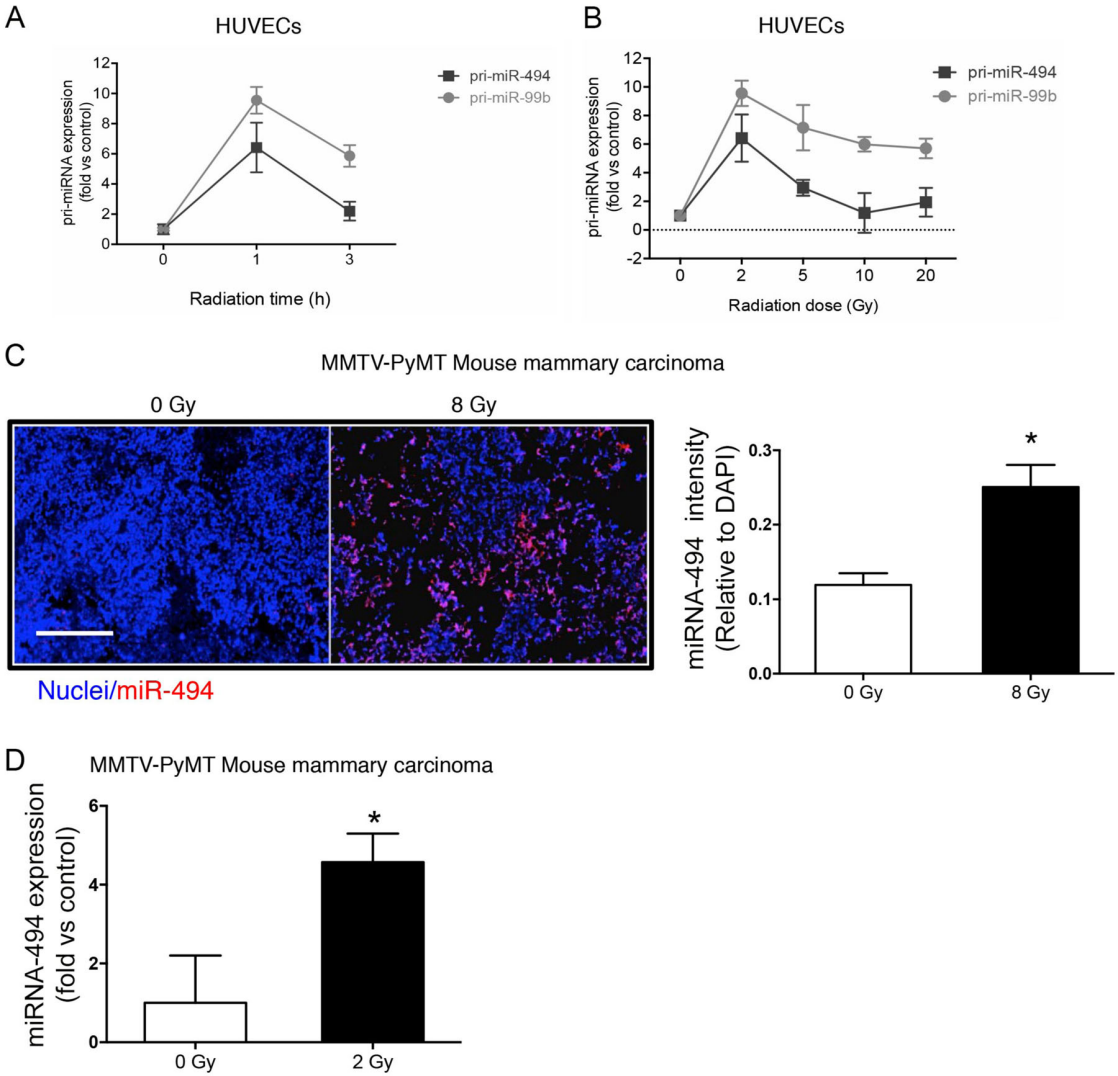
miR-494 and miR-99b are induced in response to radiation. **a** Kinetics and **b** dose response of primary miR-494 and miR-99b transcripts in HUVECs treated with the indicated dose of radiation. Kinetics was measured in response to 2 Gy. The dose response was evaluated at 1 h post radiation. **c** In situ hybridization of miR-494 on FFPE slides from PyMT-MMTV tumors irradiated with the indicated dose of radiation. Tumors were harvested 36 h post radiation. Bars depict mean + SEM of sections from 3 mice/group. miR-494 staining was normalized to the number of cells stained with DAPI. Scale bar represents 200 µm. **d** qRT-PCR of mature miR-494 from orthotopic PyMT tumor implants (*n* = 4 tumors/group) at 6 h post radiation. miR levels were normalized to U6 small RNA. Bars depict mean + SEM. *P < 0.05; two-tailed Student’s *T*-test

We previously reported a seven miR signature (miR-103, miR-494, miR-99b, miR-21, miR-224, miR-92a, and let-7a) specifically upregulated in ECs after radiation, hydrogen peroxide, and cisplatin treatment^7^. Among these, we found that miR-494 and miR-99b are both transcribed rapidly in human umbilical vein ECs (HUVECs) in response to γ-radiation with maximal induction occurring at 2 Gy (Fig. 1a, b). This induction was also observed in vivo in a genetically engineered mouse mammary carcinoma model (PyMT) via both in situ hybridization and quantitative real-time polymerase chain reaction (qRT-PCR) (Fig. 1c, d). Interestingly, in contrast to our previous observation with miR-103, miR-494 is transcribed robustly even in response to lower radiation doses and decreases to baseline by 3 h.

### Gain and loss of miR-494 affects pathological and replicative senescence

**Fig. 2:**
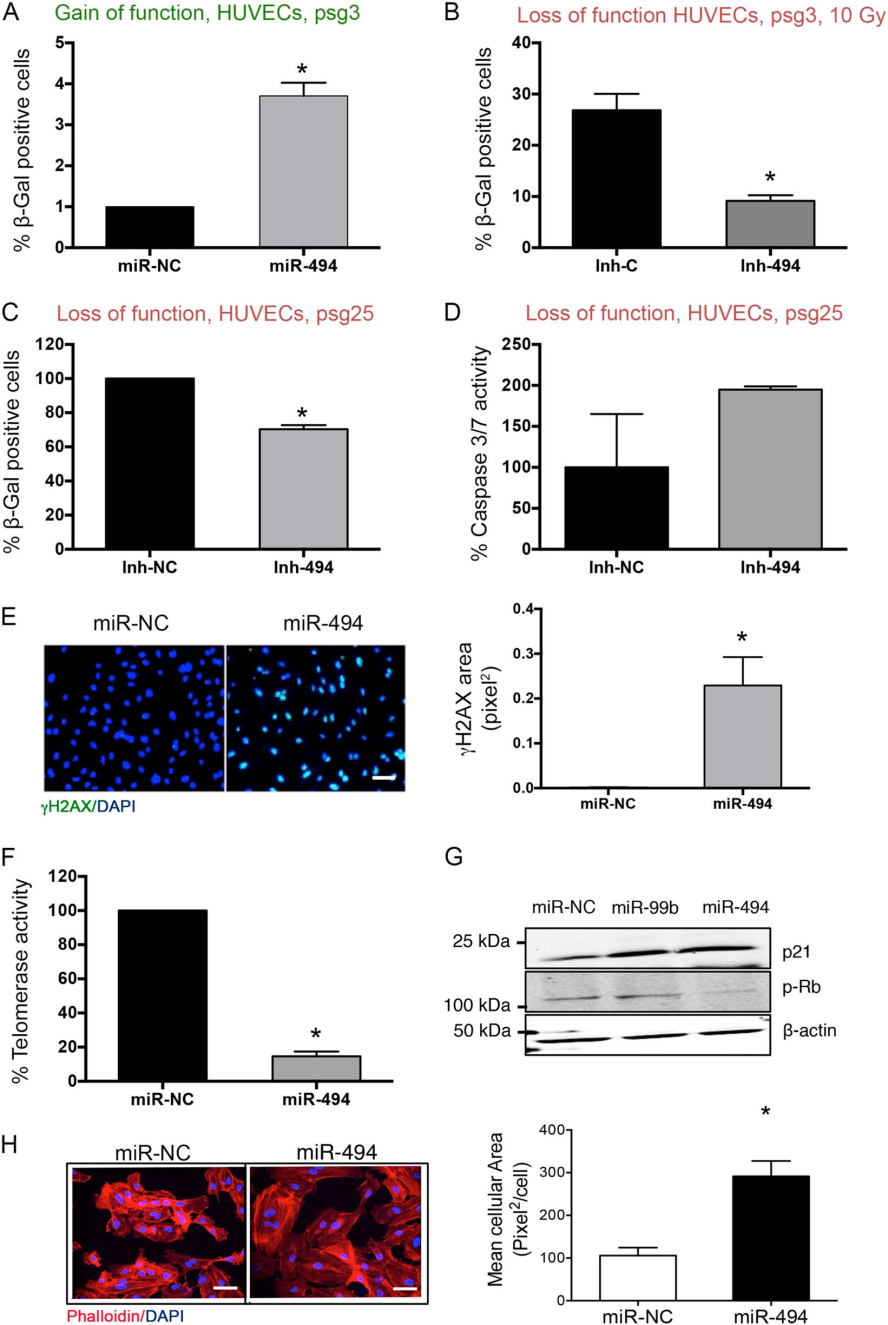
miR-494 drives pathological and physiological endothelial senescence by disrupting DNA repair. **a** SA-β-gal assay in early-passage HUVECs transfected with a miR-negative control or miR-494 mimic. Bars show % (mean + SEM) of β-gal-positive cells for at least 100 cells analyzed 48 h post transfection. **b** SA-β-gal assay at 48 h post radiation (10 Gy) of early-passage HUVECs transfected with either an inhibitor control or inhibitor miR-494. Bars show % (mean + SEM) of β-gal-positive cells for at least 100 cells analyzed. **c**, **d** SA-β-gal assay (**c**) and caspase-3/7 activity (**d**) in senescent (psg 25) HUVECs transfected with either an inhibitor control or inhibitors of miR-494 at 48 h post transfection. Bars show mean + SEM. **e** γH2AX staining in HUVECs 48 h after transfection with a miR-negative control or miR-494 mimic. Scale bar represents 50 µm. Bars depict mean γH2AX area per nuclear area + SEM of 3–4 technical replicates/group. **f** HUVECs were transfected with a miR-negative control or miR-494 mimic. After 48 h, telomerase activity was assayed using a Telo-Tagg assay. Bars depict % mean + SEM. **g** Representative western blot of senescence marker p21 and pRB in HUVECs transfected as in (**f**). **h** HUVECs were transfected with either a miR-negative control or miR-494 mimic. After 48 h, cells were fixed and stained with phalloidin (red) and DAPI (blue) as indicated. Scale bar represents 50 µm. Bars depict % mean cellular area + SEM. *P < 0.05; two-tailed Student’s *T*-test

To understand the role of these two miRs, we performed different gain- and loss-of-function assays in our EC model, HUVECs. Gain of miR-494 or miR-99b in early-passage HUVECs increased senescence-associated β-galactosidase levels (SA-β-gal) (Fig. 2a and Supplementary Figure 1). In contrast, inhibition of miR-494 and to a lesser extent, inhibition of miR-99b, decreased SA-β-gal levels significantly in HUVECs undergoing pathological senescence induced by a high dose of radiation (Fig. 2b and Supplementary Figure 1). Similarly, inhibition miR-494 in late-passage senescent HUVECs decreased their SA-β-gal and increased activation of caspase-3 and -7 (Fig. 2c, d). Based on the robustness of the phenotype, we chose to focus further experiments on miR-494. We observed miR-494-induced increase in SA-β-gal in human microvascular ECs (HMVECs) and normal human lung fibroblasts (NHLFs) suggesting this phenotype was not exclusive to venous endothelium (Supplementary Figure 2). Given the effects on senescence, we asked if miR-494 affected cell cycle progression. We found that ectopic expression of miR-494 increased G1 arrest, whereas the inhibition of the miR decreased the G2 arrest in response to a 10 Gy dose of radiation (Supplementary Figure 3).

Consistent with the known role of DNA damage in cellular senescence, miR-494 expression increased histone H2AX phosphorylation de novo (Fig. 2e). In addition, we also observed miR-494 impacted other hallmarks of senescence—a decrease in telomerase activity (Fig. 2f), an increase in the cell cycle regulator p21 as well as a decrease in Rb hyperphosphorylation (Fig. 2g). Moreover, phalloidin staining in HUVECs after 48 h of miR-494 treatment also revealed flattened and multinucleated cells (Fig. 2h), another morphological phenotype associated with stress-dependent senescence. Taken together, these observations establish that miR-494 drives EC senescence likely via pathways involved in DNA damage or repair.

### miR-494 targets the MRN DNA repair complex affecting senescence

**Fig. 3:**
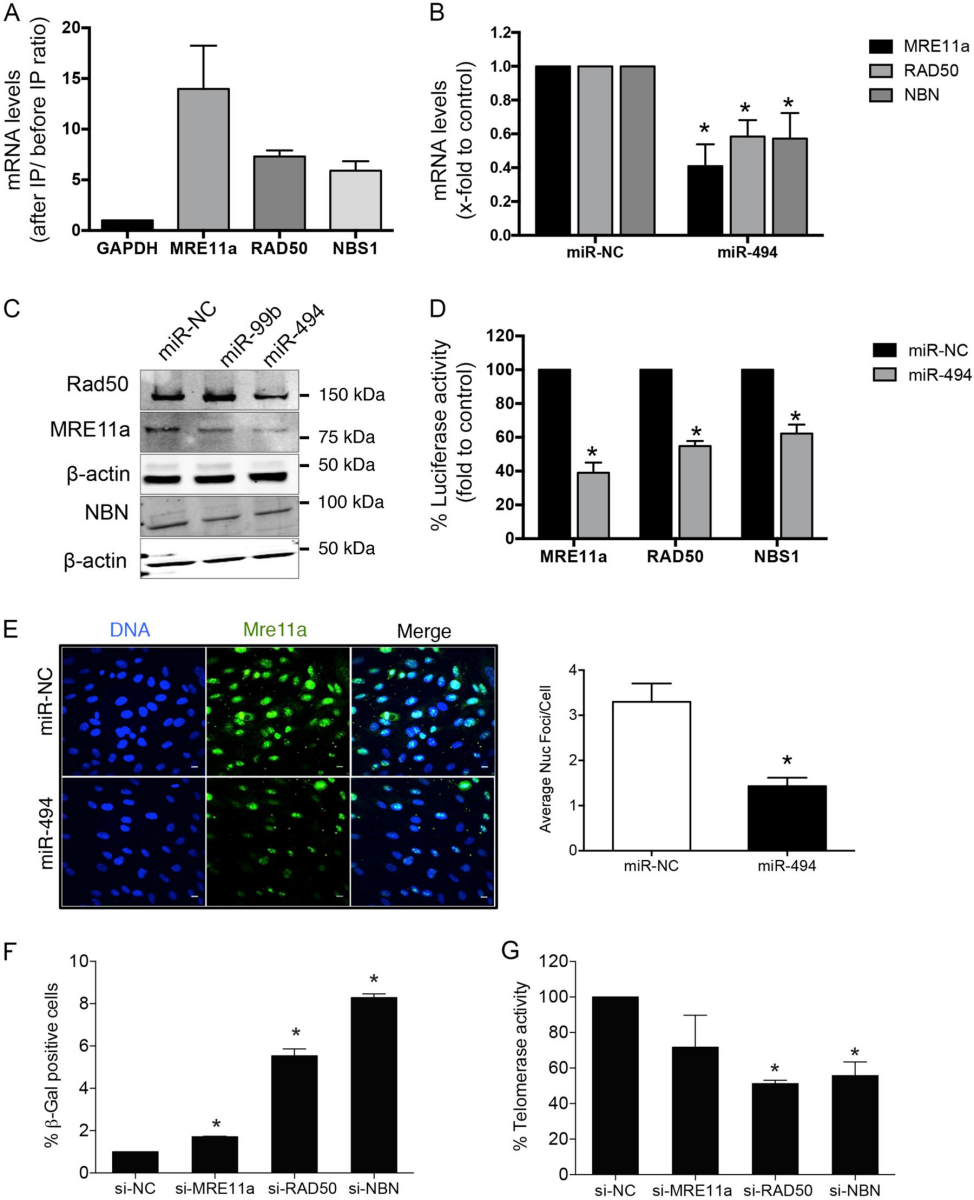
miR-494 targets the MRN complex in ECs. **a** miR-TRAP immunoprecipitation. Bars depict mean + SEM of mRNAs bound to the RISC complex quantified by qRT-PCR in HEK-293T cells transfected with miR-494 for 24 h. **b** qRT-PCR depicting target mRNA levels in HUVECs transfected with either miR-negative control or miR-494. Bars show mean + SEM of the three MRN complex members. **c** Representative western blot of *MRE11a*, *RAD50*, and *NBN* after 48 h transfection of indicated miRs in HUVECs. **d** Luminescence from 3’-UTR-luciferase constructs for *MRE11a*, *RAD50*, and *NBN* 24 h after transfection with miR-494. Graph represents mean + SEM of three independent experiments. **e** HUVECs were plated on glass coverslips and 24 h later transfected with either miR-494 or miR-negative control. After 48 h, cells were fixed and stained with *MRE11a* antibody. Scale bar represents 10 µm. Bar graph depicts average foci/ cell + SEM. **f** β-Gal assay and **g** telomerase activity in HUVECs transfected for 48 h with specific siRNAs against *MRE11a*, *RAD50*, and *NBN*, or an siRNA-negative control. Bars depict % mean + SEM. *P < 0.05; two-tailed Student’s *T*-test

To identify the possible targets of miR-494 we used a DNA damage gene signature array and compared genes suppressed by both miR-494 and miR-99b (Supplementary Figure 4). Interestingly, we identified three common target genes for both miRs: *MRE11a*, *RAD50*, and *NBN*. Using RNA hybrid we mapped putative binding sites for these miRs on all three target 3’-untranslated regions (3’-UTRs) (Supplementary Figures 5-6). miR-494 directly bound MRE11A mRNA, and to a lesser extent, *RAD50* and *NBN* mRNAs, as measured by a miR-Trap assay (Clontech) (Fig. 3a). Transfection of miR-494 decreased both MRN RNA (Fig. 3b) and protein levels (Fig. 3c). On the other hand, miR-99b transfection decreased MRN RNA levels but only had a modest effect on the MRN protein levels (Supplementary Figure 7 and Fig. 3c). Moreover, miR-494 decreased activity of a luciferase reporter cloned upstream of each 3’-UTR of the MRN complex genes (Fig. 3d). Finally, miR-494-transfected HUVECs also showed a decrease in *MRE11a* nuclear foci (Fig. 3e) as measured by immunostaining.

miRs typically regulate several target mRNA transcripts making their functional attribution challenging^19–23^. To address this, we used a target protector, a locked nucleic acid (LNA) stabilized oligonucleotide, that binds completely to a specific miR-binding site on a single target mRNA, to rescue it from the negative regulation of the miR. We confirmed that the MRE11A target protector rescued MRE11A RNA levels in the miR-494-transfected cells compared to a control target protector oligo (Supplementary Figure 8A). Functionally, the MRE11A target protector significantly restored the telomerase activity and to a lesser extent, the SA-β-gal levels in miR-494-transfected cells (Supplementary Figure 8B-C). We then sought to determine if disruption of the MRN complex using siRNAs (Supplementary Figure 9) recapitulated the phenotypes observed with the miR. Indeed, silencing each of the components of the MRN complex induced senescence and impaired telomerase activity (Fig. 3f, g). Similarly, siRNAs against MRN, as well as Mirin-1, a small-molecule inhibitor specific to *MRE11a*^24^, also enhanced senescence in HMVECs (Supplementary Fig 10). These data indicate that the miR-494 downregulates the MRN machinery and thereby exacerbates DNA damage and drives senescence.

### The MRN pathway cross talks with angiogenic growth factors

**Fig. 4:**
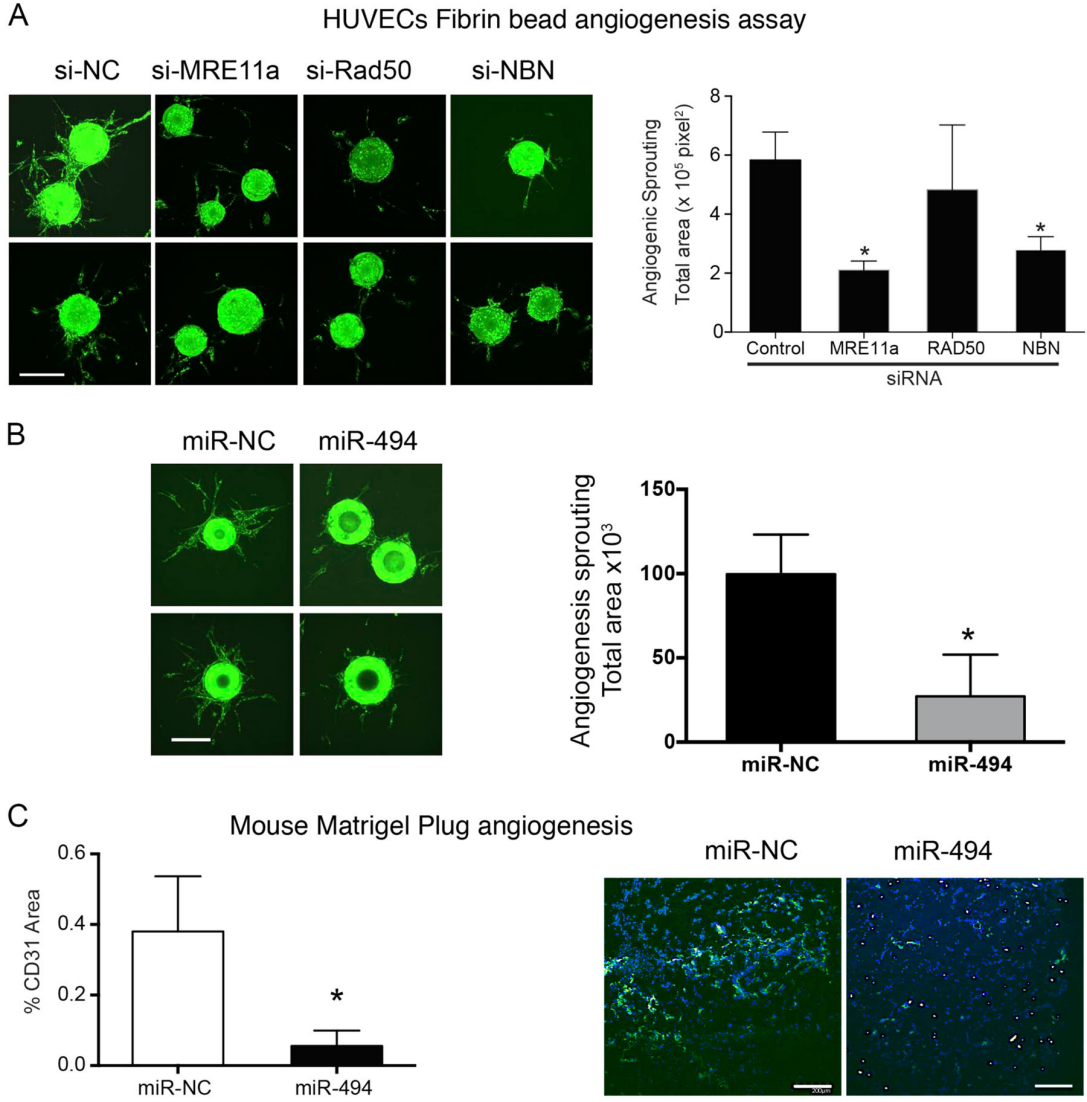
MRN pathway is necessary for sprouting angiogenesis. **a** Fibrin bead angiogenesis assay. HUVECs were transfected with the indicated siRNAs and assessed for their sprouting angiogenesis potential. The images show representative lectin-stained beads (green) for each condition. Bars depict mean + SEM of lectin area analyzed across at least 25 beads/group. *P < 0.05; two-tailed Student’s *T*-test. **b** Fibrin bead angiogenesis assay. HUVECs were transfected with the indicated miRs and assessed for their sprouting angiogenesis potential. The images show representative lectin-stained beads for each condition. Bars depict mean + SEM of lectin area analyzed across at least 25 beads/group. **c** CD31 staining in vivo. bFGF-containing Matrigel plugs were implanted subcutaneously in nude mice and treated with miR-NC or miR-494 in vascular-targeted 7C1 nanoparticles on days 5 and 6. Mice were sacrificed at day 7 and plugs were harvested for tissue sections. Angiogenesis was measured by staining sections with anti-CD31 (green) and DAPI (blue). Scale bar represents 200 µm. Quantification of CD31 area from at least 3 mice/group is shown. Bars show mean + SEM

Disruption of DDR in ECs has been shown to modulate angiogenesis via VEGF^3,4^. Therefore, we asked if targeting MRN also influences VEGF signaling in ECs. Indeed, combinatorial disruption of both VEGFR2 and *MRE11a* with Mirin-1 and Vandetanib, a small-molecule inhibitor for VEGFR2, led to a decrease in proliferation of HUVECs with a synergistic interaction at the lowest dose (Chou-Talalay combination index < 0.4) (Supplementary Fig 11A). Mirin-1 treatment affected basic fibroblast growth factor (bFGF)-mediated pERK signaling and to a lesser extent VEGF-induced pERK in HUVECs (Supplementary Fig 11B). Surprisingly, knockdown of VEGFR2 diminished the senescence-promoting activities of both the siRNAs targeting the MRN and Mirin-1 (Supplementary Fig 11C, D). These experiments argue that the disruption of MRN could inhibit angiogenic growth factor signaling. Next, we asked if this effect of reduced growth factor signaling translated into deficient angiogenic sprouting. Indeed, siRNA targeting of Mre11a or *NBN* or miR-494 significantly decreased angiogenic sprouting in a three-dimensional (3D) sprouting angiogenesis assay^25^ (Fig. 4a, b). Similar results were obtained with miR-99b in this model (Supplementary Fig 12).

Having observed the effects of targeting this pathway via miRs, small molecules, and siRNAs on in vitro angiogenesis, we sought to determine if this strategy would impact angiogenesis in vivo. We first used a growth factor-induced angiogenesis model by implanting bFGF-containing Matrigel plugs in mice. We treated mice with either a miR-494 mimic or a control mimic in vascular-targeted 7C1 nanoparticles^7,26^. We found a robust decrease in CD31 staining in miR-494-treated plugs compared to the control mimic-treated group (Fig. 4c).

### miR-494 mimic disrupts growth factor-driven and pathological angiogenesis

**Fig. 5:**
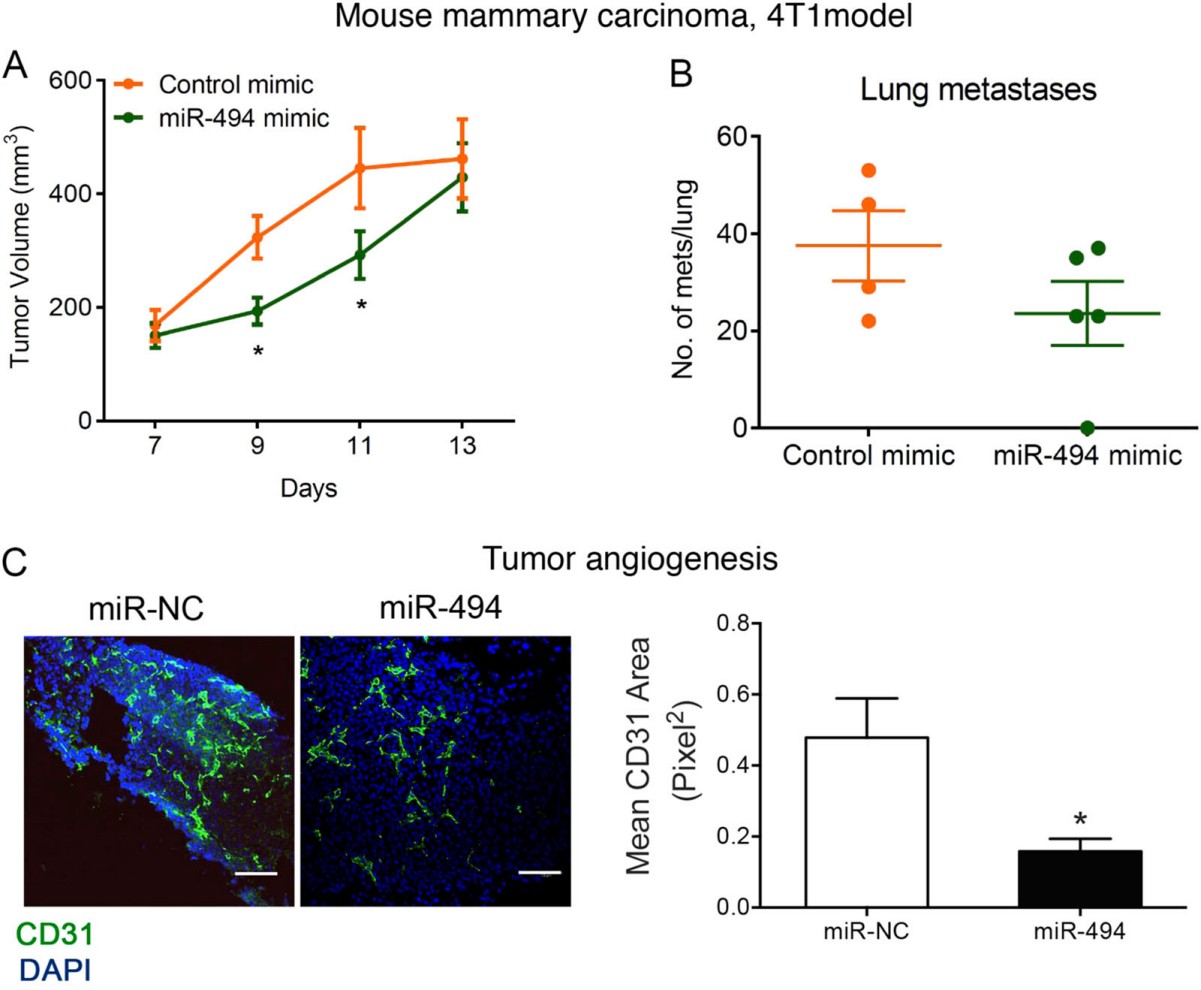
miR-494 disrupts angiogenesis in vitro and in vivo. **a** 4T1 mouse mammary carcinoma cells (1 × 10^4^) were implanted in the mammary fat pads of 6- to 8-week-old female Balb/C mice (*n* = 4–5/group). Mice were randomized to receive either control miR mimic or miR-494 mimic formulated as miR-7C1 nanoparticles (0.7 mg/kg, i.v.) on days 12, 14, and 16. Primary tumor volume measurements (**b**) and gross metastatic foci per lung on day 20 (**c**) are shown. Error bars depict SEM. *P < 0.05 by Mann–Whitney *U*-test. **d** Angiogenesis was measured by staining the 4T1 tumor sections with anti-CD31 (green). Scale bar represents 200 µm. Bars show CD31 area normalized the tumor area as mean + SEM from *n* = 3 tumors/group. *P < 0.05; two-tailed Student’s *T*-test

To test the utility of miR-494 vascular-targeted 7C1 nanoparticles in a pathological angiogenesis model, we utilized a highly aggressive, triple-negative 4T1 mouse mammary carcinoma model. Treatment of vascularized tumors with miR-494 in the 7C1 nanoparticles resulted in a delayed tumor growth for a few days (Fig. 5a). We did not observe a significant impact on lung metastases (Fig. 5b). However, we observed the miR-494-treated tumors had a significant decrease in angiogenesis as measured by CD31 staining (Fig. 5c). Our in vivo studies indicate that miR-494 is able to decrease both growth factor-induced and pathological angiogenesis in vivo.

DNA damage in ECs increases expression of miR-494. miR-494 leads to senescence by targeting the DNA repair complex MRN and decreases angiogenesis. Our results demonstrate that the DNA repair pathway, specifically the MRN complex has a functional role in angiogenesis and we propose that we can target this pathway for the development of novel anti-angiogenic therapies.

## Discussion

ECs in different pathophysiological conditions such as the tumor vasculature, an atherosclerotic artery, or a diabetic retina suffer DNA damage. While the role of DNA repair in cell cycle and cell death pathways is well appreciated, there are only two studies that implicate DNA repair proteins as regulators of angiogenesis^3,4^. Based on a surprising observation that miR-processing enzyme Dicer impacts EC DNA repair, we set up a screen and identified a group of seven miRs that are induced by DNA damage. While we showed that miR-103 enhanced EC death and decreased angiogenesis, the role of the other miRs in the context of EC DNA damage and angiogenesis was not clear. Here we have identified that miR-494 and to a lesser extent miR-99b, decrease the MRN complex and drive EC senescence resulting in decreased angiogenesis.

miR-494 has been shown to be highly upregulated in retinoblastoma^27^, cardiovascular pathologies such as cardiac injury^28^, and in atherosclerotic lesion development^29^. miR-494 has also been reported as an angiogenesis inhibitor, supporting our data here^30–32^. Esser et al.^33^ demonstrated that miR-494 is downregulated in ECs treated with the pro-angiogenic factor BMP4, in opposition to an increase in the pro-angiogenic miR-126. miR-494 has been implicated in tumor senescence and the development of resistance to radiation and chemotherapy^33–36^.

We find that miR-494 targets the MRN complex to affect EC senescence and sprouting angiogenesis. EC senescence leads to dysfunction in cardiovascular diseases^37,38^. Loss-of-function mutations in the MRN genes cause inherited genetic disorders that are characterized by elevated sensitivity of patients’ cells to radiation damage^39,40^. Interestingly, there is some evidence that *NBN* disruption has an anti-angiogenic effect in vivo^41^.

While the role of MRN in DSB repair is well appreciated, there is an emerging literature demonstrating functions beyond DNA repair. For example, decreased *MRE11a* has been shown to increase T-cell aging in arthritis due to compromised telomere maintenance and heterochromatin unraveling^42^. Recent structural studies also shed light on how the phosphorylation of NBS influences its interaction with TRF2 and dictates the repair of telomeres^43^. *RAD50* downregulates the association of TRF1 from telomeres and also contribute to maintain telomere length^44^. We saw a decrease in telomerase activity across miRs and siRNA experiments targeting the MRN complex. Moreover, the *MRE11a* target protector partially restored this function, implying this was an important miR-494 target responsible for the compromised telomerase activity. Therefore, we suspect that the telomere maintenance function of the MRN complex drives the EC senescence downstream of miR-494 and miR-99b induced by DNA damage.

Our observations indicate that the function of these miRs in regulating telomerase activity and EC senescence may depend on the expression of VEGFR2. This is surprising, since expression of VEGFR2 may steer cells into a robust replication program rather than a cell cycle exit characteristic of senescence. However, it is possible that replication stress and DNA damage are enhanced in the presence of these miRs. Similarly, our findings demonstrate a decrease in MRN either by miR-99b induction or Mirin-1 affects the VEGFR signaling at the level of pERK. These data argue that the MRN pathway and the VEGFR2 signaling cross-talk functions as a threshold to determine the level of DNA damage in ECs. In the presence of significant DNA damage the MRN pathway is disrupted and the VEGF signaling is diminished thereby decreasing angiogenesis. It is possible that during physiological aging, increase in miR-494 and miR-99b levels render the ECs less responsive to VEGF levels and therefore, decrease angiogenic responses. We envision that this senescence-associated decrease in VEGF sensitivity functions in a feedforward loop and contributes to cardiovascular aging. Data from our mouse models demonstrate the utility of exploiting this pathway to decrease pathological angiogenesis. On the basis of these studies, we propose that the MRN complex is novel anti-angiogenic target.

Our previous work^7^ and data in this manuscript showcase three stress-dependent miRs in ECs (miR-103, miR-494, and miR-99b) target DNA repair pathways and affect angiogenesis. Our ongoing studies indicate the expression and function of these miRs may be coordinated. For instance, silencing of the cell cycle regulator p53 and Chk2 inhibited basal expression of some of these miRs, whereas silencing the antioxidant transcription factor Nrf2 decreased basal and radiation-induced expression (data not shown). Functionally, these miRs target a critical subset of the DDR network (Supplementary Figure 13) and decrease angiogenesis in vitro and in vivo. Elucidating the function of these miRs and broadly, the DDR pathway, in angiogenesis will enable us to understand how the vasculature handles genotoxic stress and provide new avenues for the development of therapeutics across a spectrum of diseases.

## Materials and methods

### Cell culture and reagents

HUVECs and HMVECs (Lonza) were cultured in EGM-2 media (Lonza) supplemented with 10% fetal calf serum (Hyclone). NHLFs (Lonza) were cultured in FBM media (Lonza) supplemented with 10% fetal calf serum (Hyclone). 4T1 cells (American Type Culture Collection) were culture in McCoy’s media, Dulbecco’s modified Eagle’s medium, or RPMI-1640 supplemented with 10% fetal calf serum and antibiotics. Cells were tested and found negative for mycoplasma contamination before use in the assays described. Mirin-1 and Vandetanib were purchased from Cayman Chemical and Selleckchem, respectively. VEGF was purchased from PeproTech, Inc.

### miRs/anti-miRs/siRNAs

miR mimics, inhibitors, and respective controls were purchased from Life Technologies and Exiqon. For in vivo studies, high-performance liquid chromatography-purified miRs were purchased from Life Technologies in bulk quantities. siRNAs against *MRE11a*, *RAD50*, and *NBN* were purchased from Life Technologies. VEGFR2 Gapmer and *MRE11a* target protector were purchased from Exiqon.

### Vectors/plasmids

*MRE11a* luciferase-3-UTR plasmid was purchased from SwitchGear Genomics. *RAD50* and *NBN* luciferase constructs were generated by cloning the entire 3’-UTR regions into pmiR-REPORT vector (Ambion). Luciferase assay reagents were purchased from SwitchGear Genomics and Promega.

### Transfection

Cell were transfected at 50–60% confluence using standard forward transfection protocols using RNAimax reagent (Life Technologies) for miRs, siRNAs, or gapmers, and Lipofectamine 2000 for plasmid or plasmid RNA dual transfections. Typically 50 nM RNA and 1–2 μg plasmid DNA were used for transfections. Target protectors were transfected at a concentration of 50 nM or equivalent to the miR amounts.

### Radiation of cells/mice

Cells or mice were irradiated on a Shepherd 137cesium irradiator at a rate of ~166 cGy/min. In tumor-targeted radiation experiments, mice were restrained in a lead shield with flank cut-outs (Brain tree scientific) to minimize exposure to the non-tumor areas.

### β-Gal senescence assay

HUVECs were transfected for 48–72 h with the microRNAs or siRNAs. After this time cells were washed with cold phosphate-buffered saline (PBS) and then stained for β-galactosidase activity following the manufacturer’s protocol (Senescence Cells Histochemical Staining Kit, Sigma).

### Telomerase activity assay

Cells were transfected with microRNAs or siRNAs for 24 h. Cells were lysed and processed according to the manufacturer´s instructions (Quantitative Telomerase Detection Kit, Allied Biotech). The telomerase activity level in the cell extract was determined through its ability to synthesize telomeric repeats onto an oligonucleotide substrate. The resultant extended product was subsequently amplified by polymerase chain reaction (PCR).

### Cell Titer-Glo/Caspase Glo

Cells were transfected in a six-well plate with miR mimics, siRNAs, or inhibitors, and the corresponding controls from Life Technologies as previously described^7^. Cell Titer-Glo and Caspase 3/7 Glo were analyzed at 48–96 h post treatments, according to the manufacturer’s instructions.

### Western blot and densitometric analysis

After treatment, cells were washed in PBS and lysed in RIPA buffer (Sigma) supplemented with Complete Protease inhibitor cocktail (ROCHE) and phosphatase inhibitors cocktail 2 and 3 (Sigma). Lysed cells were harvested by scraping, and proteins were analyzed by western blot. Equivalent amounts of protein were loaded on a 4–12% gradient SDS-polyacrylamide gel (BioRAD) and transferred for 30 min in a TransBlot turbo (BioRAD) onto nitrocellulose membranes. Membranes were blocked in 5% milk or 3% bovine serum albumin (BSA) and incubated with antibodies as indicated: *MRE11a* (Cell Signaling, 4847, 1:1000); *RAD50* (Cell Signaling, 3427, 1:1000); NBS1 (Cell Signaling, 14956, 1:500); p21 (Cell Signaling, 2947, 1:1000); and pRb (Cell Signaling, 9301, 1:500). β-actin (Sigma, A5316, 1:10,000 1 h RT) was used as a housekeeping control for the total levels of protein loaded. Membranes were washed in Tris-buffered saline (TBS) with Tween 20 and incubated with secondary antibodies from Licor Biosciences. Licor antibodies used were Goat anti-Mouse 925–68020 (1:15 000) and Goat anti-Rabbit 925–32211 (1:15 000). Blots were scanned on the Licor Odyssey scanner according to the manufacturer’s instructions.

### RNA extraction, RT-PCR, miR profiling

Total RNA and microRNA were isolated using a miRVana microRNA isolation kit (Ambion). Reverse transcription was performed using TaqMan™ Advanced cDNA Synthesis Kit (Life Tech) according to the manufacturer’s instructions. RT-PCR was performed using multiplexed TaqMan primers (Applied Biosystems). The relative quantification of gene expression was determined using the 2^−ΔΔCt^ method^35^. Using this method, we obtained the fold changes in gene expression normalized to an internal control gene, *GAPDH* or *U6 snRNA*, respectively. For target analysis, a 92-gene DNA damage array (Life tech, 4418773) was used and for senescence phenotype profiling, the Human Cellular Senescence array was utilized (SA Biosciences) per the manufacturer’s recommendations.

### γH2AX staining

In all, 100 000 HUVECs were cultured on glass coverslips in 24-well plates and transfected with miRs/siRNAs using RNAimax (Life Technologies). Cells were fixed at different time points with 4% paraformaldehyde for 10 min at room temperature, permeabilized with 90% methanol for 10 min at 4 °C. Coverslips were blocked with 1.5% normal goat serum (NGS) and incubated with primary antibody H2AX (Abcam, 11174, 1:500), at a 1:1000 dilution in NGS for 1 h, washed and then incubated with secondary antibody for 30 min, washed and then mounted on glass slides for confocal imaging.

### miR in situ hybridization

In situ hybridization was performed on frozen tumor sections as described in our previous studies^45,46^ using a digoxigenin (DIG)-labeled miR-494 LNA probe (Exiqon). DIG was detected by an anti-DIG horseradish peroxidase antibody (Roche) and amplified using a TSA-Plus Cy3 system (Perkin Elmer).

### miR-TRAP/RISC TRAP assay

293T cells were co-transfected with a plasmid coding for a flag-tagged dominant negative GW418 mutant (Clontech kit #632016) along with a control mimic, miR-99b, or miR-494 mimic according to kit instructions. After 24 h, the RNA protein complexes were crosslinked and the RISC complex was immunoprecipitated using an anti-FLAG antibody and RNA was isolated for qRT-PCR of target genes. The fold enrichment was calculated using pre and post IP controls as well as normalization to the control mimic pull-downs.

### 3D angiogenic sprouting assay

Early-passage HUVECs were coated on cytodex-3 beads (GE Healthcare) at a density of 10 million cells/40 μl beads and incubated in suspension for 3–4 h with gentle mixing every hour. They were plated on Tissue Culture (TC)-treated six-well dishes overnight and resuspended in a 2 mg/ml fibrin gel with 200 000 human smooth muscle cells. The gel was allowed to polymerize and complete EGM-2 media was added. Sprouts were visualized from days 3 to 4 via confocal imaging after overnight incubation with fluorescein isothiocyanate-labeled *Ulex europaeus* lectin (Vector labs). Immunofluorescence imaging was performed on a Yokogawa CSU-W1 spinning disk confocal microscope with ×20, 0.45 Plan Fluor objective (Nikon).

### Cell cycle analysis

HUVECs were transfected for 48 h with microRNAs, inhibitors, or siRNAs. Cells were then harvested, washed, and fixed in 70% ice-cold ethanol at 4 °C overnight. Then, cells were centrifuged, washed with cold PBS, and re-centrifuged. Cells were then resuspended in 250 µl PBS and stained with 10 µl propidium iodide (1 mg/ml) and 10 µl RNase A (10 mg/ml) for 30 min at room temperature. DNA content was assessed using flow cytometry (CANTO II) to calculate the percentage of cells in subG1, G0/G1, S, and G2/M phases with FlowJo software.

### Immunofluorescence and microscopy

In some experiments, CD31a and *MRE11a* were visualized using immunofluorescence staining from Optimal Cutting Temperature (OCT) sections. Slides were fixed with 4% paraformaldehyde and stained overnight for CD31 488 (BD Bioscience 611986 1:200 o/n), *MRE11a* (Cell Signaling Technology 1:100), and Phalloidin Alexa 647 (1:50). For *MRE11a* antibody, following day fixed cells were incubated with Goat anti-Rabbit Alexa 488 (1:500) in 5%BSA/TBS for 1 h. Imaging was performed on a Nikon Spectral C1 confocal microscope (Nikon C1si with EZC1 acquisition software, Nikon Instruments) with Plan Apo ×10/0.45 air, Plan Apo ×20/0.75 air, and Plan Apo ×60/1.40 oil objective lenses (Nikon). Some immunofluorescence imaging was performed on a Yokogawa CSU-W1 spinning disk confocal microscope with 20 0.45 Plan Fluor objective (Nikon). All images were taken with a channel series. Images were analyzed with ImageJ software for quantitation.

### pERK ELISA

We measured the abundance of pERK with an ELISA kit (PathScan^®^ Phospho-p44/42 MAPK (Thr202/Tyr204) Sandwich ELISA, Cell Signaling Technology) following the manufacturer’s instructions.

### In vivo assays

All animal work was approved by the OHSU Institutional Animal Use and Care Committee. Immune-compromised 8- to 10-week-old female nu/nu mice were purchased from Jackson Labs. Growth factor-reduced Matrigel (BD) with 400 ng/ml recombinant human bFGF (Millipore) was injected subcutaneously in nu/nu mice. Mice were injected intravenously (i.v.) with 7C1 nanoparticles containing miR-494 or control miR (~1 mg/kg, i.v) 3 or 4 days after plugs were implanted. At day 7 mouse tissues were harvested and processed to obtain RNA or frozen in OCT for tissue staining. 4T1 cells (1 × 10^4^) were implanted into the mammary fat pad #4 of 6- to 8-week-old female Balb/C mice in 100 μl Matrigel. Mice were randomized into groups once the average tumor volume reached 150 mm^3^, approximately 10 days after implantation. Mice were treated with 7C1 nanoparticles containing miR-494 or control miR (0.7 mg/kg, i.v.). Mice were euthanized ~day 18–20 for analysis of metastatic burden in lungs. Additional experiments were carried out in postpubertal female FVB/n mice (10–12 weeks), which received injections of 50 000 PyMT-derived tumor cells into the right lower mammary fat pad for induction of orthotopic tumors as described in ref. ^47^.

### Statistics

All statistical analysis was performed using Excel (Microsoft) or Prism (GraphPad). Two-tailed Student’s *T*-test was used to calculate statistical significance when groups were normally distributed. For more than two groups, two-way analysis of variance was used. For data that were not normally distributed, Mann–Whitney *U*-test was used. Variance was similar between treatment groups.

## Electronic supplementary material

Supplementary Figures 1-13

## References

[1] Bautista-Nino PK, Portilla-Fernandez E, Vaughan DE, Danser AH, Roks AJ (2016). DNA damage: a main determinant of vascular aging. Int. J. Mol. Sci..

[2] Regina C (2016). Vascular ageing and endothelial cell senescence: molecular mechanisms of physiology and diseases. Mech. Ageing Dev..

[3] Economopoulou M (2009). Histone H2AX is integral to hypoxia-driven neovascularization. Nat. Med..

[4] Okuno Y, Nakamura-Ishizu A, Otsu K, Suda T, Kubota Y (2012). Pathological neoangiogenesis depends on oxidative stress regulation by ATM. Nat. Med..

[5] Guo Z, Kozlov S, Lavin MF, Person MD, Paull TT (2010). ATM activation by oxidative stress. Science.

[6] Kang HT (2017). Chemical screening identifies ATM as a target for alleviating senescence. Nat. Chem. Biol..

[7] Wilson R (2016). MicroRNA regulation of endothelial TREX1 reprograms the tumour microenvironment. Nat. Commun..

[8] Williams RS, Williams JS, Tainer JA (2007). Mre11-Rad50-Nbs1 is a keystone complex connecting DNA repair machinery, double-strand break signaling, and the chromatin template. Biochem. Cell Biol..

[9] Lafrance-Vanasse J, Williams GJ, Tainer JA (2015). Envisioning the dynamics and flexibility of Mre11-Rad50-Nbs1 complex to decipher its roles in DNA replication and repair. Prog. Biophys. Mol. Biol..

[10] Dimitrova N, de Lange T (2009). Cell cycle-dependent role of MRN at dysfunctional telomeres: ATM signaling-dependent induction of nonhomologous end joining (NHEJ) in G1 and resection-mediated inhibition of NHEJ in G2. Mol. Cell. Biol..

[11] Porro A, Feuerhahn S, Lingner J (2014). TERRA-reinforced association of LSD1 with MRE11 promotes processing of uncapped telomeres. Cell Rep..

[12] Ju YJ (2006). Decreased expression of DNA repair proteins Ku70 and Mre11 is associated with aging and may contribute to the cellular senescence. Exp. Mol. Med..

[13] Gao R, Singh R, Kaul Z, Kaul SC, Wadhwa R (2015). Targeting of DNA damage signaling pathway induced senescence and reduced migration of cancer cells. J. Gerontol. A Biol. Sci. Med. Sci..

[14] Boon RA, Dimmeler S (2015). MicroRNAs in myocardial infarction. Nat. Rev. Cardiol..

[15] Santulli G (2016). MicroRNAs and endothelial (Dys) function. J. Cell. Physiol..

[16] Fish JE, Srivastava D (2009). MicroRNAs: opening a new vein in angiogenesis research. Sci. Signal..

[17] Shi H, Li P, Liang W, Chen J, Gao Y (2010). Mechanisms of microRNA-mediated regulation of angiogenesis. Front. Biosci..

[18] Landskroner-Eiger S, Moneke I, Sessa WC (2013). miRNAs as modulators of angiogenesis. Cold Spring Harb. Perspect. Med..

[19] Bartel DP (2009). MicroRNAs: target recognition and regulatory functions. Cell.

[20] Ebert MS, Sharp PA (2012). Roles for microRNAs in conferring robustness to biological processes. Cell.

[21] Osella M, Bosia C, Corá D, Caselle M (2011). The role of incoherent microRNA-mediated feedforward loops in noise buffering. PLoS Comput. Biol..

[22] Li X, Cassidy JJ, Reinke CA, Fischboeck S, Carthew RW (2009). A microRNA imparts robustness against environmental fluctuation during development. Cell.

[23] Inui M, Martello G, Piccolo S (2010). MicroRNA control of signal transduction. Nat. Rev. Mol. Cell Biol..

[24] Garner KM, Pletnev AA, Eastman A (2009). Corrected structure of mirin, a small-molecule inhibitor of the Mre11-Rad50-Nbs1 complex. Nat. Chem. Biol..

[25] Nakatsu MN (2003). Angiogenic sprouting and capillary lumen formation modeled by human umbilical vein endothelial cells (HUVEC) in fibrin gels: the role of fibroblasts and Angiopoietin-1☆. Microvasc. Res..

[26] Dahlman JE (2014). In vivo endothelial siRNA delivery using polymeric nanoparticles with low molecular weight. Nat. Nanotechnol..

[27] Zhao JJ (2009). Identification of miRNAs associated with tumorigenesis of retinoblastoma by miRNA microarray analysis. Childs Nerv. Syst..

[28] Wang X (2010). MicroRNA-494 targeting both proapoptotic and antiapoptotic proteins protects against ischemia/reperfusion-induced cardiac injury. Circulation.

[29] Wezel A (2015). Inhibition of microRNA-494 reduces carotid artery atherosclerotic lesion development and increases plaque stability. Ann. Surg..

[30] Asuthkar S (2014). Irradiation-induced angiogenesis is associated with an MMP-9-miR-494-syndecan-1 regulatory loop in medulloblastoma cells. Oncogene.

[31] Chen S (2015). MicroRNA-494 inhibits the growth and angiogenesis-regulating potential of mesenchymal stem cells. FEBS Lett..

[32] Welten SM (2014). Inhibition of 14q32 MicroRNAs miR-329, miR-487b, miR-494, and miR-495 increases neovascularization and blood flow recovery after ischemia. Circ. Res..

[33] Esser JS (2017). Bone morphogenetic protein 4 regulates microRNAs miR-494 and miR-126-5p in control of endothelial cell function in angiogenesis. Thromb. Haemost..

[34] Weng JH (2016). miR-494-3p induces cellular senescence and enhances radiosensitivity in human oral squamous carcinoma cells. Int. J. Mol. Sci..

[35] Ohdaira H, Sekiguchi M, Miyata K, Yoshida K (2012). MicroRNA-494 suppresses cell proliferation and induces senescence in A549 lung cancer cells. Cell. Prolif..

[36] Comegna M (2014). Identification of miR-494 direct targets involved in senescence of human diploid fibroblasts. FASEB J..

[37] Minamino T (2002). Endothelial cell senescence in human atherosclerosis. Role Telomere Endothel. Dysfunct..

[38] Kurz DJ (2004). Chronic oxidative stress compromises telomere integrity and accelerates the onset of senescence in human endothelial cells. J. Cell Sci..

[39] Dzikiewicz-Krawczyk A (2008). The importance of making ends meet: mutations in genes and altered expression of proteins of the MRN complex and cancer. Mutat. Res..

[40] O’Malley BW, Li D, Carney J, Rhee J, Suntharalingam M (2003). Molecular disruption of the MRN(95) complex induces radiation sensitivity in head and neck cancer. Laryngoscope.

[41] Araki K (2010). Molecular disruption of NBS1 with targeted gene delivery enhances chemosensitisation in head and neck cancer. Br. J. Cancer.

[42] Li Y (2016). Deficient activity of the nuclease MRE11A induces T cell aging and promotes arthritogenic effector functions in patients with rheumatoid arthritis. Immunity.

[43] Rai R (2017). NBS1 phosphorylation status dictates repair choice of dysfunctional telomeres. Mol. Cell.

[44] Wu Y, Xiao S, Zhu XD (2007). MRE11-*RAD50*-NBS1 and ATM function as co-mediators of TRF1 in telomere length control. Nat. Struct. Mol. Biol..

[45] Anand S (2010). MicroRNA-132-mediated loss of p120RasGAP activates the endothelium to facilitate pathological angiogenesis. Nat. Med..

[46] Kelley KA (2017). Understanding and resetting radiation sensitivity in rectal cancer. Ann. Surg..

[47] Shiao SL (2015). TH2-polarized CD4( + ) T cells and macrophages limit efficacy of radiotherapy. Cancer Immunol. Res..

