# Supplementary material for: MicroRNA regulation of the MRN complex impacts DNA damage, cellular senescence, and angiogenic signaling
Source: Cell Death Dis. 2018 May 24;9(6):632. doi: 10.1038/s41419-018-0690-y (PMC5967305; doi:10.1038/s41419-018-0690-y)
Supplement: Supplementary file 1 — Supplementary Figures 1-13 [file 41419_2018_690_MOESM1_ESM.pdf]

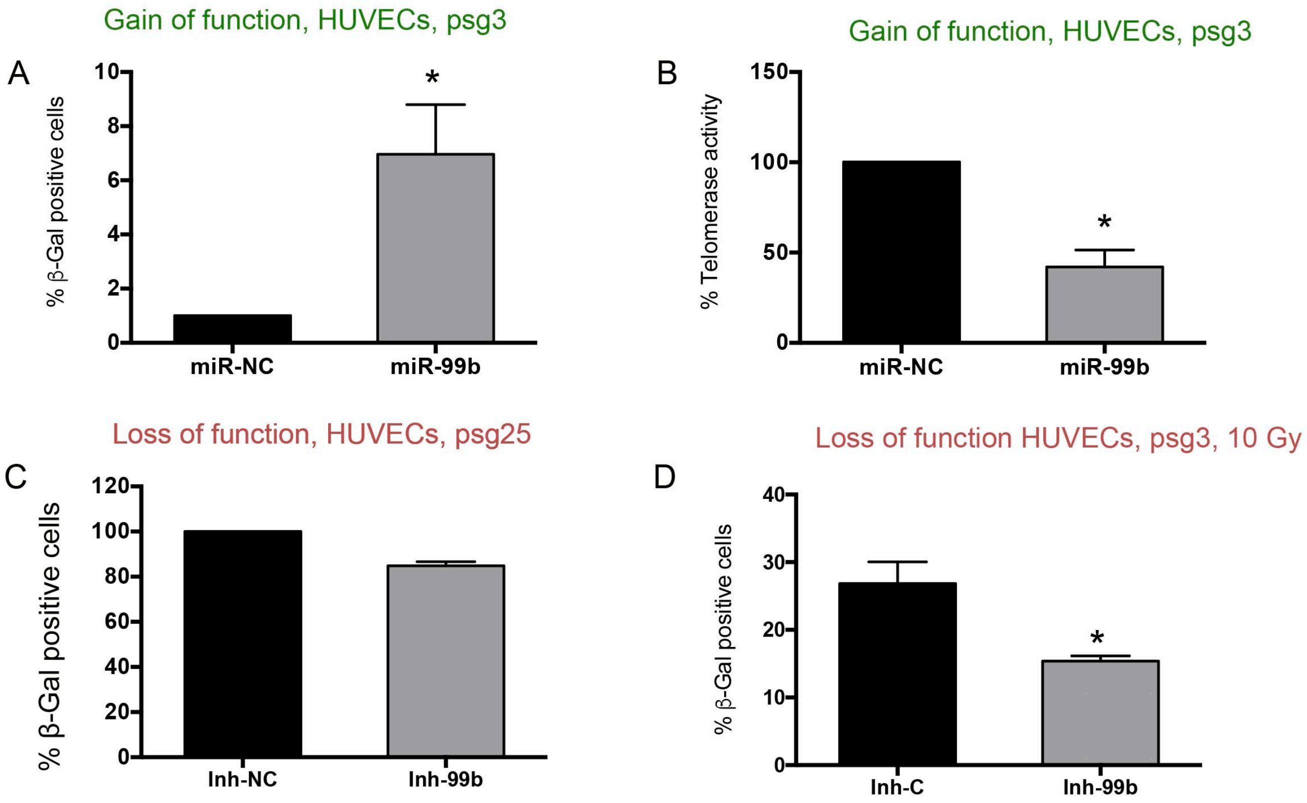

Supplementary Figure1. miR-99b drives pathological and physiological endothelial senescence by disrupting MRN complex.
A) SA-β-Gal assay in early passage HUVECs transfected with a miR negative control or miR-99b mimic. Bars show % (mean + SEM) of β-gal positive cells for at least hundred cells analyzed 48h post transfection. **B)** HUVECs were transfected with a miR negative control or miR-99b mimic. 48h later telomerase activity was assayed using a Telo-Tagg assay. Bars depict % mean + SEM. **C)** SA-β-Gal assay in senescent (psg 25) HUVECs transfected with either an inhibitor control or inhibitors of miR-99b at 48h post transfection. Bars show mean + SEM. **D)** SA-β-Gal assay at 48h post radiation of early passage HUVECs transfected with either an inhibitor control or inhibitor miR-99b. Bars show % (mean + SEM) of β-gal positive cells for at least hundred cells analyzed.

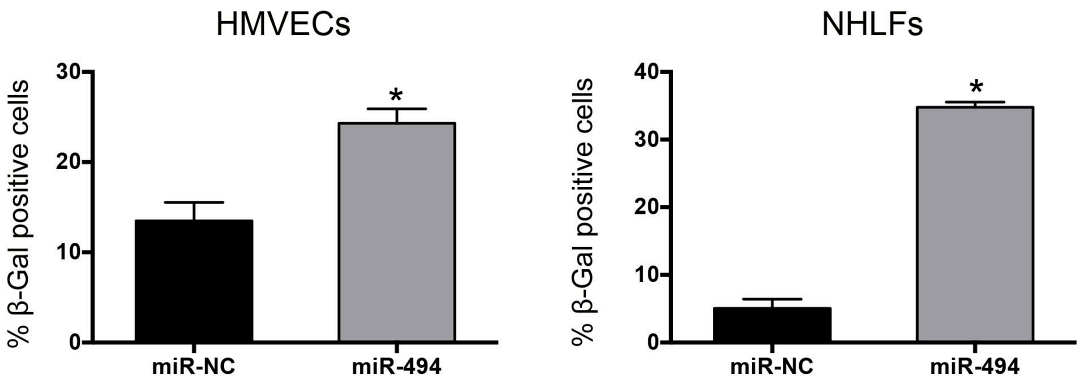

Supplementary Figure 2. Senescence phenotype in other normal cells.
β-Gal assay in HMVECs or NHLFs transfected for 48 hours with miR-NC or miR-494. Bars show % mean + SEM of β-gal positive cells for at least hundred cells analyzed.

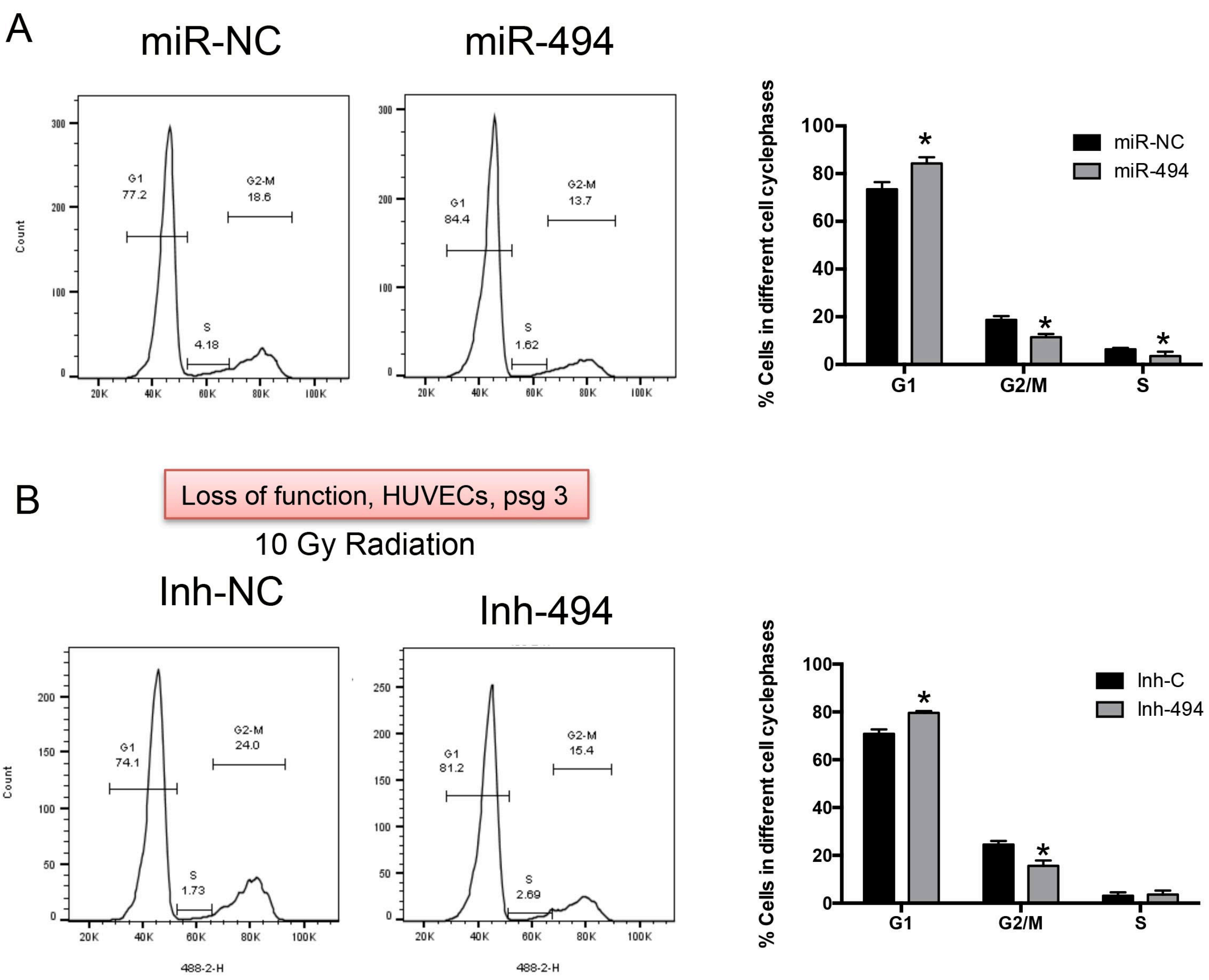

Supplementary Figure 3. Gain and loss of senescence miRs affects cell cycle progression.
HUVECs were transfected with the indicated miR mimics (A) or inhibitors (B) and cell cycle analysis was performed via flow cytometry of propidium iodide stained cells at 48h post transfection (A). Transfected cells were irradiated at 24h and cell cycle analysis was performed at 48h post radiation (B). Bars show % mean + SEM of 3 independent experiments. P*< 0.05, two-way anova.

Gene	miR-99b	miR-494
APEX1	0.64	1.00
ATM	0.77	0.87
ATR	0.72	0.50
BARD1	0.66	0.30
BRCA1	0.23	0.52
BRCA2	0.52	0.54
CCNO		1.01
CHECK1	0.37	0.52
CHECK2	2.58	0.75
DCLRE1A	1.11	0.90
ERCC1	1.36	0.86
ERCC2	2.74	1.06
ERCC3	0.57	0.74
ERCC4	0.42	0.72
ERCC5	0.97	0.66
ERCC6	0.19	0.51
ERCC8	1.03	0.97
FANCA	0.99	0.83
FANCC	2.34	0.58
FANCD2	0.23	0.63
FANCE	2.38	1.04
FANCF	1.19	0.63
FANCG	1.69	0.76
FEN1	1.67	0.69
GADD45A	0.75	0.75
GADD45B	2.13	1.18
GADD45G	1.12	0.53
GTF2H1	0.26	0.55
GTF2H3	0.47	0.70
HUS1	0.80	0.66
IGF1		
LIG1	3.74	0.96
LIG3	0.54	0.80
LIG4	0.61	0.77
MAPK10		
MAPK11	8.37	1.39
MAPK12	2.51	0.98
MAPK14	0.33	0.78
MAPK8	0.36	0.60
MAPK9	0.32	0.64
MBD4	0.48	0.61
MDM2	0.49	1.24
MGMT	3.48	0.88
MRE11A	0.25	0.44
MSH2	0.30	0.69
MSH3	0.63	0.53

Gene	miR-99b	miR-494
MSH6	0.52	0.80
NBN	0.24	0.36
NTHL1	4.00	1.07
OGG1	2.50	1.38
PARP1	0.37	0.70
PCNA	0.54	0.82
PNKP	3.23	1.43
POLA1	1.03	0.77
POLB	0.93	0.67
POLD1	13.99	0.62
POLG	2.08	0.95
POLH	0.60	0.87
POLK	0.47	0.41
POLQ	0.44	0.30
POLR1B	0.35	0.90
POLR1C	1.19	0.92
POLR2A	1.32	0.69
POLR2B	0.15	0.57
POLR2C	0.85	1.15
PRKDC	0.19	0.66
PSMA3	0.99	0.50
PSMB10	4.91	0.80
PSMB5	2.19	0.73
PSMB8	5.85	0.84
PSMB9	0.57	0.47
PSMC4	0.68	0.73
RAD1	0.77	0.72
RAD17	0.73	0.77
RAD23B	0.24	0.66
RAD50	0.31	0.49
RAD51	0.70	0.58
RAD52	0.70	0.60
RAD9A	1.15	0.91
RPA2	0.92	0.69
RPA3		
SMUG1	5.70	1.12
TP53	0.76	0.92
TREX1	0.70	0.54
TREX2		1.12
XAB2	1.37	0.99
XPA	1.02	1.05
XPC	0.64	1.05
XRCC1	0.69	0.67
XRCC4	0.44	0.73
XRCC5	0.26	0.63
XRCC6	1.03	0.66

Numbers indicate fold change vs GAPDH

Supplementary Figure 4. Identification of miR targets by targeted transcriptional profiling.

Heat map depicting mRNA changes induced by miR-99b and miR-494 in HUVECs at 24h post transfection as analyzed by a TaqMan Human DNA Repair qRT-PCR gene signature array.

4021 GCACGGTGGCTCACGCCTGTGATCCTAGCACTTTGGGAGGCCAAAGCAGGCGGATCACCT
4081 GAGGTCAGGAGTTCGAGACCAGCAGGGCCAACATGGCGAAACCCCGACTCTACTAAAAAT
4141 ACAAAAATTAGCCAGGCATGGTGGTGGGCGCCTGTAATCCCAACTACTCAGGAGGCTGAG
4201 GCAGGAGAATTGCTTGAACCTGGGAGTTGGAGGTCACGGTGAGCTGATATCACGCCATTG
4261 CACTCCAGCCTGGGCAACAGAGCAAGACTCTGTCTCAATCAATCTATCCATCAATCGATA
4321 AGAACCCCAGATTGTATAGCTACATGTTTTAGCCCCCTTTTCAAAGTATATGTTCTCCTT
4381 GGTACTTATTTTGACATTCTGACTTTTCTACATATGCTTTATCAACCTCTTAATTAAACC
4441 ATCATTGTCTATTTTGAGAGATAACTGCGCTGCTTCCCGTTGTGTGTTTTAAATGTTATT
4501 GTTCAGTTTGAGTCAAATAAAAGGATATTTAATCTGTGGTGCCTTGA

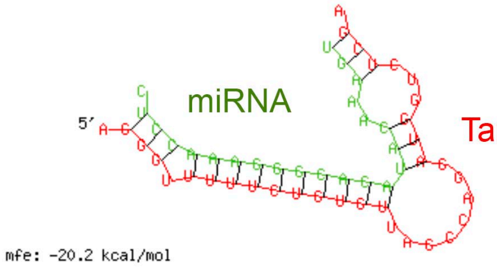

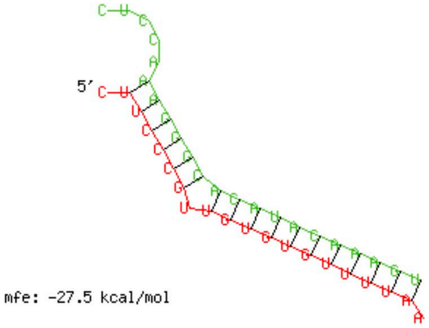

B

1801 TTGCAAGTTAAAACAGACTTCCCAATTTTAAATCTGGTTTCCCCCTGAATATGTGGCATC
1861 CTTGGCAGCACTTCTGAGAGTGGCTGCTTTCATTCCAAGAAGCCCATGGGTTTGGAGGTG
1921 GGATAGGTGCCTTTCTGGCTTCTCATTGCTGCTTCTAGATCAGTCTCCAAATATCCCCCT

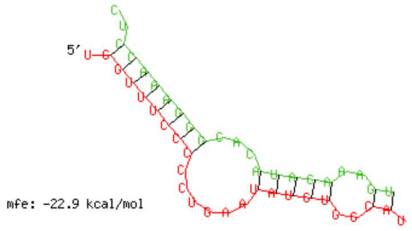

C

1621 TTTTCCTTTTTAACCATTCCAATCGGTGTGTAGTGATGTTTCATTTTGGTTTTAATTTGT
1681 ATATCCCTGATAGCTATAATTGGGTCATAGAAATTCTTTATACATTCTAGATGCAAGTCT
1741 CTTGTCGGATATATGTATTGAGATATTACACCTAGTCTGTGGCTTGACTGTTTTCTTTAT

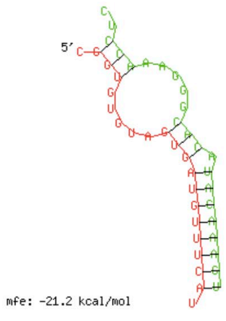

Supplementary Figure 5: RNA hybrid models for miR-494

A. mRNA sequence of human MRE11a, **B.** RAD50 and **C.** NBN. Binding sites are highlighted in red. MRE11a-miR-494 target blocker was designed against the site highlighted in red and underlined.

A

1561 ACCTACCCCTGCACAAGCCATGCTGGCTCAGTCTGAGCTGTGGGCCACATCAGCTAGTGG
1621 CTCTTCTCATGCATCAGTTAGGTGGGTCTGGGTGAGAGTTATAGTGAGGGAATGGTCACT
1681 AAAGTATCCTGACAAGTTCCTAGGAAAAAAGGAATAAAGTTTTTTTCCTTAAAAAAAAAA
1741 AAATTGCTCTTGGCTGTGAAAAGAGGTACTAAATGCGATTCAGTTCACCGCTAAGGAAAG
1861 AATACTATGTATCAAGCCCTGAAAATATGGTAAATAAAACGTGACAGGGAAACCTTTTTT
1921 TGATTGAATATTGTTACATAGTTAAATGTGCTATATATCCTTAATATTTTATATTGATCC
1981 TGCAAAATCTGTTGGTTTTAGGGGAGTTTTGTTTTTTGTTTCTAACAATTTTCAGACCTG
2041 TTGGTATAGGAATGTAGAAGTCTTTCAGATGATTTGAAAGCAGCTGCATTTGCTCTTGGA

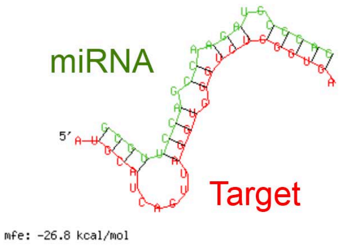

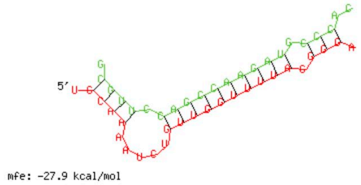

B

1261 CTAGAACCCGTGACTGTTACTTTATACAGCAAAGGAAACTTTGCAGATGTGATTAAAGCT
1321 AAGGACCTTAAGACAGAGTATCCTGGGGGTGGTGGTGGGGTGGGGGGGGTCCTAAATGT
1381 AATCACGAGTAAGATTAAGAGCAAATCAATTCTAGTCATATATTAAACATCCACAATAAC
3601 GTAGTCTCAGCTACTTGGCTGAGATAGGATCCCTGGAGTCTGAGGGTTCAAGGCTGCAGT
3661 GAGCTGTGATTTGTGCCACTGCACCCCAGCCTGGGTGGGTGGCAGAGTGAGACCCTGTCT
3721 AAAAAGACAAAAAAAAAAAAAAAAAAAAAGCAGCAGCAGCTGAAAGTTGGCAGGAGCACA

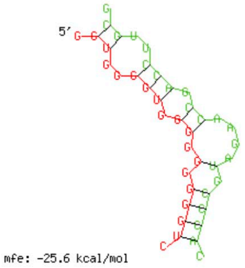

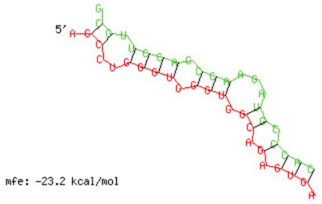

C

661 TTTTCACCAGAATGGAAAGACCTGTACCCCTTTTTGGTGGTCTTACTGAGCTGGGTGGGT
721 GTCTGTTTTGAGCTTATTTAGAGTCCTAGTTTTCCTACTTATAAAGTAGAAATGGTGAGA
781 TTGTTTTCTTTTTCTACCTTAAAGGGAGATGGTAAGAAACAATGAATGTCTTTTTTCAAA
1141 CAGCTGCAAGGGAGTTAGGGAAATGAAGGTCTTTTTTTAAAAGCTTCTCAGCCTTCCTAG
1201 GGAACAGAAATTGGGTGAGCCAATCTGCAATTTCTACTACAGGCATTGAGACCAGTTAGA
1261 TTATTGAAATATTATAGAGAGTTATGAACACTTAAATTATGATAGTGGTATGACATTGGA

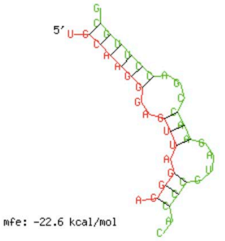

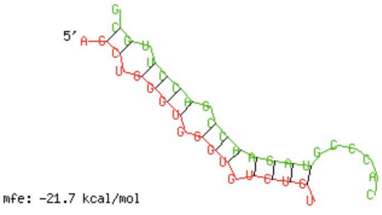

Supplementary Figure 6. RNA hybrid models for miR-99b **A.** mRNA sequence of human MRE11a **B.** RAD50 and **C.** NBN. Binding sites are highlighted in red.

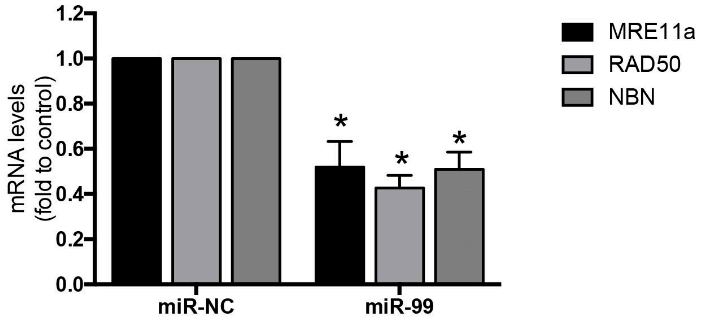

Supplementary Figure 7. miR-99b targets the MRN complex in ECs.
qRT-PCR depicting target mRNA levels in HUVECs transfected with either miR-negative control or miR-99b. Bars show mean + SEM of the three MRN complex members.

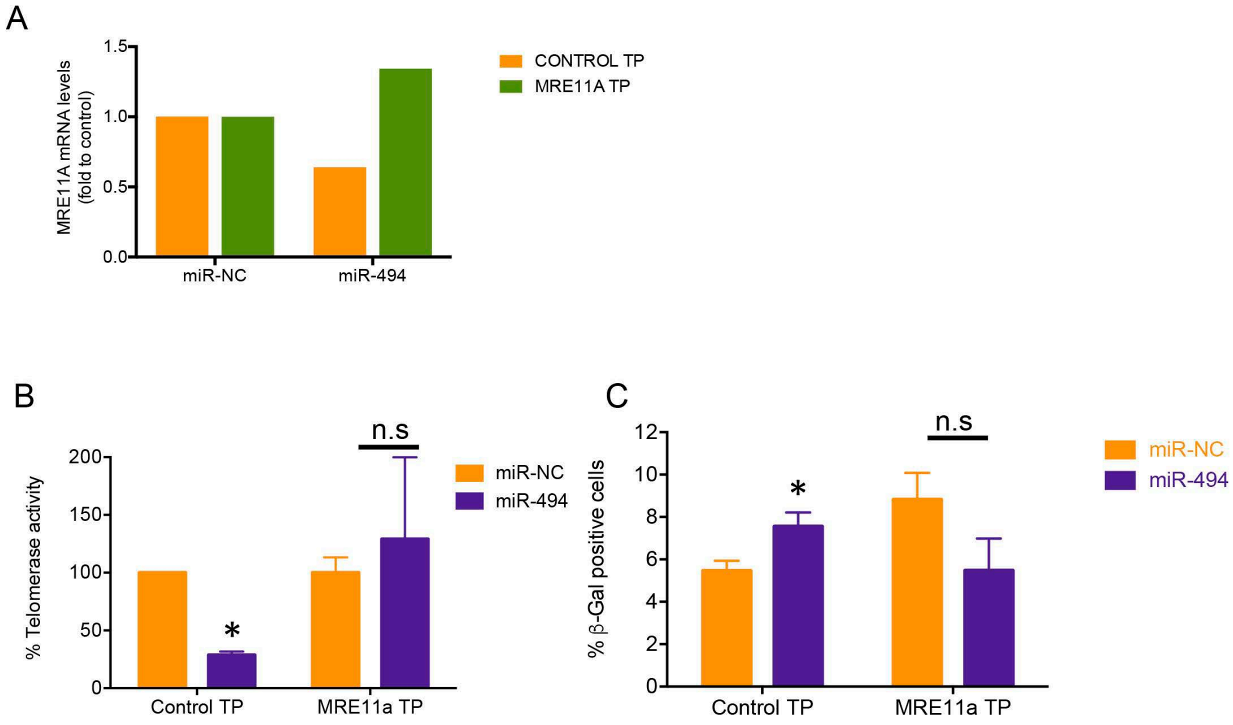

Supplementary Figure 8. Protecting Mre11a from miR-494 rescues telomerase activity and decreases senescence.

A. HUVECs were co-transfected with a target protector for miR-494 binding site in MRE11a-3´-UTR or a scrambled control target protector, and the corresponding miR. Bar graph depicts mean +SEM of Mre11a. **B-C**. HUVECs were transfected as described in A) for 48h and telomerase activity (B) and senescence associated β-Gal (C) was assayed as described in Fig 2. Bars depict % mean + SEM of Telomerase activity in B) and show % mean + SEM of β-gal positive cells for at least hundred cells analyzed in C).

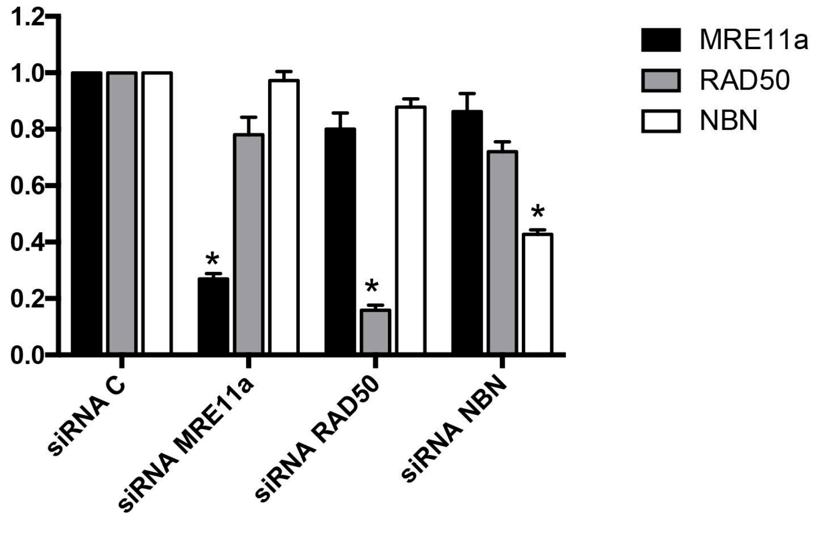

Supplementary Figure 9. siRNA validation.
HUVECs were transfected with either a negative control siRNA or specific siRNAs against MRE11a, RAD50 and NBN and qRT-PCR was performed at 24h for the indicated target genes.

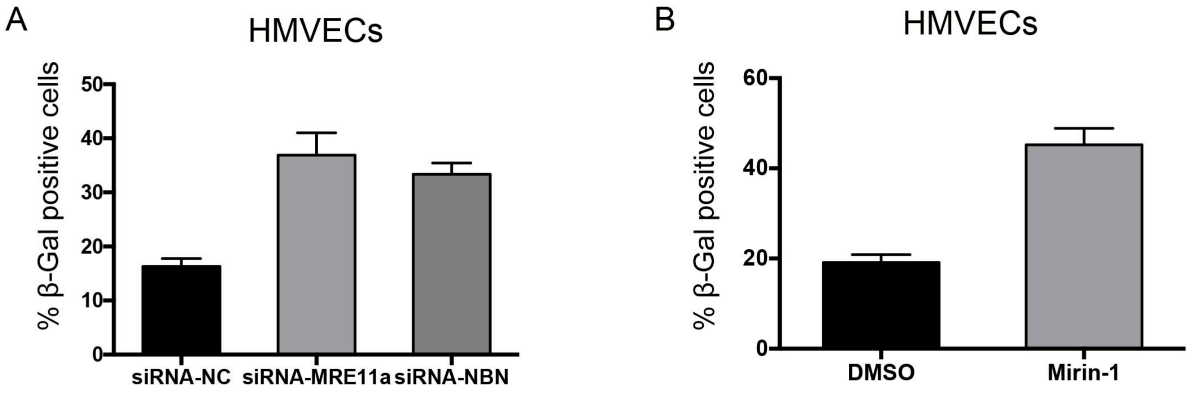

Supplementary Figure 10. Senescence phenotype upon MRN disruption in HMVEC cells.
β-Gal assay in HMVECs transfected for 48 hours with **A**. siRNA-NC, siRNA-MRE11a or siRNA-NBN or **B**. treated with Mirin-1 (50 μM) for 24h. Graphs show % mean ± SEM of β-gal positive cells for at least hundred cells analyzed.

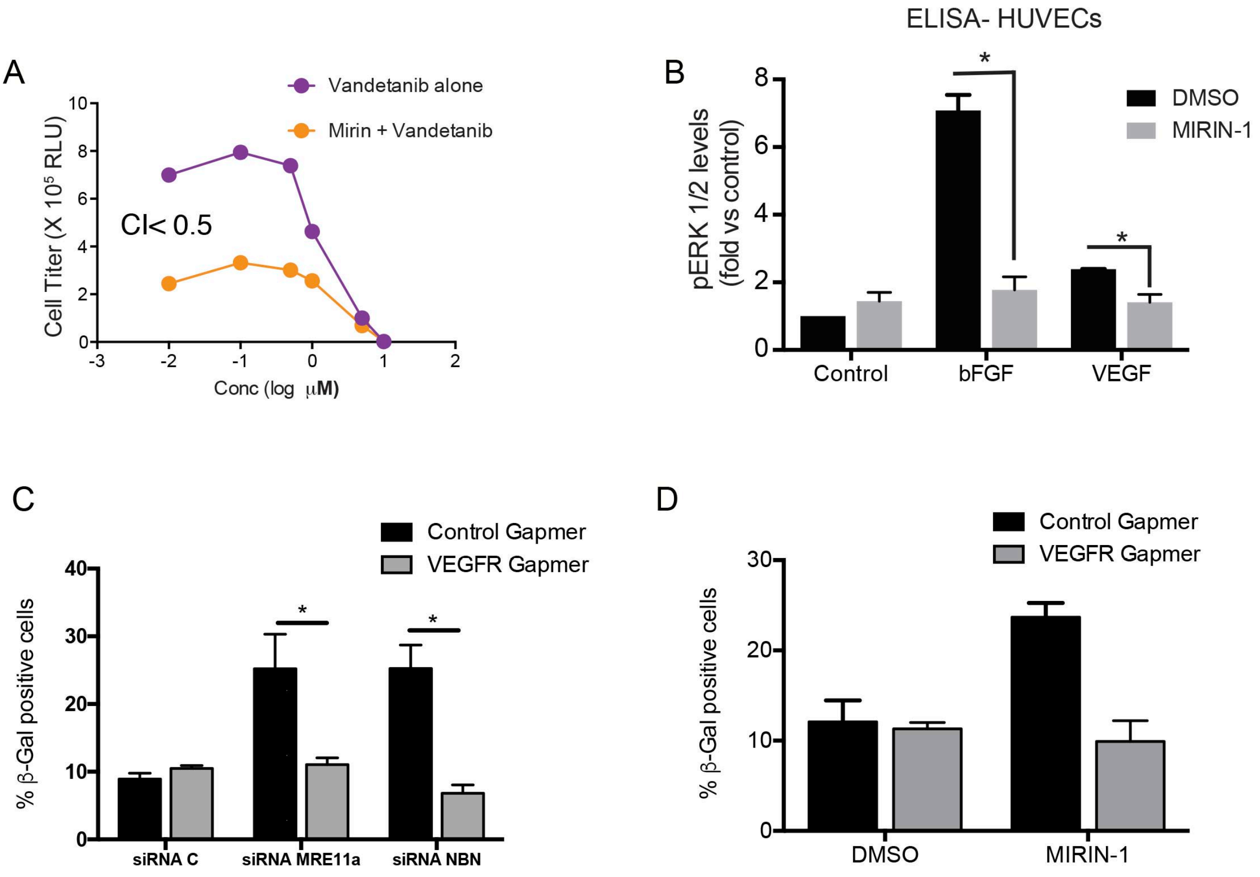

Supplementary Figure 11. MRN pathway interacts with angiogenic signaling
A) Cell proliferation assay in HUVECs treated with VEGFR2 inhibitor Vandetanib (10 μM) alone or in combination with Mirin-1 (50 μM). Combination Index (CI) at the lowest concentration is shown. **B)** Representative pERK ELISA. HUVECs were starved overnight and treated with Mirin-1 (50 μM). 30 mins later cells were treated for 10 min with VEGF (50ng/ml) or bFGF (100ng/ml) and p-ERK levels were measured using an ELISA assay. Graphs depict mean p-ERK levels + SEM. **C)** β-Gal assay in HUVECs co-transfected for 48h with siRNAs against MRE11a or NBN in combination with a VEGFR2 silencing gapmer. Bars show % mean + SEM of β-gal positive cells for at least hundred cells analyzed. **D)** β-Gal assay in HUVEC transfected for 48h with VEGFR2 gapmer and treated with Mirin-1 (50μM) for 24h. Bars show % mean ± SEM of β-gal positive cells for more than 100 hundred cells analyzed.

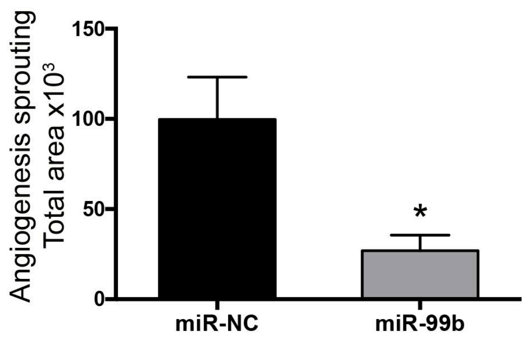

Supplementary Figure 12. miR-99b inhibits sprouting angiogenesis.
Fibrin bead angiogenesis assay. HUVECs were transfected with miR-99b and assessed for their sprouting angiogenesis potential. Bars depict mean +SEM of lectin area analyzed across at least 25 beads per group.

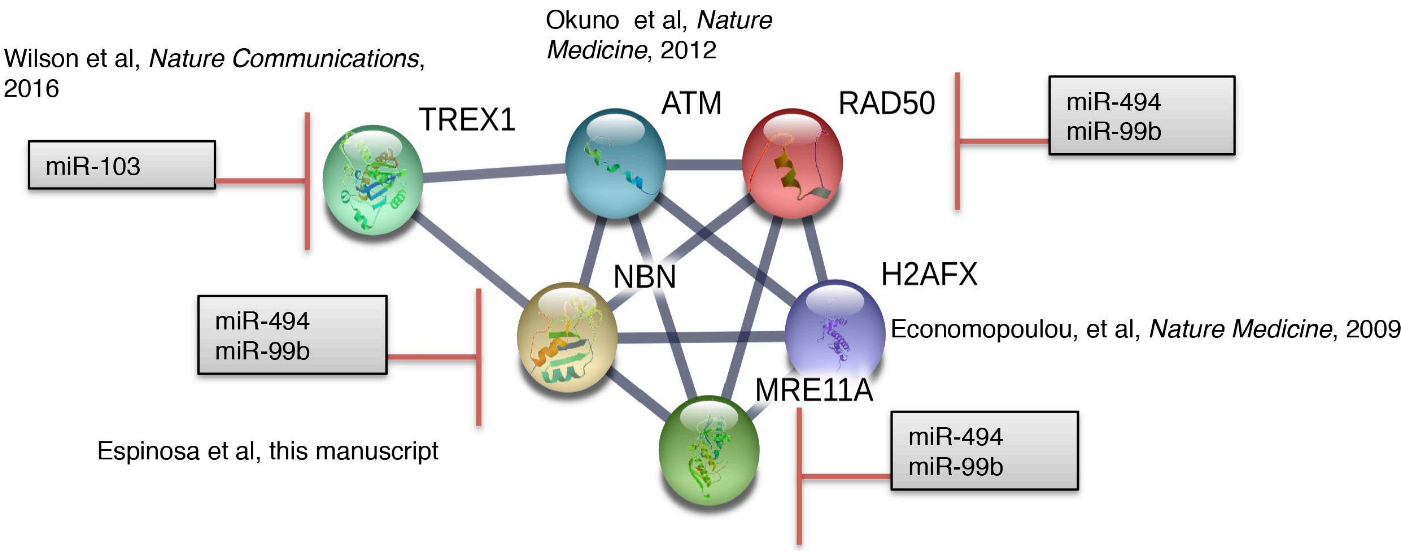

Supplementary Figure 13. DNA damage repair network drives endothelial stress responses and angiogenesis
